# An efficient method for the prediction of deleterious multiple-point mutations in the secondary structure of RNAs using suboptimal folding solutions

**DOI:** 10.1186/1471-2105-9-222

**Published:** 2008-04-29

**Authors:** Alexander Churkin, Danny Barash

**Affiliations:** 1 Department of Computer Science, Ben-Gurion University, 84105 Beer Sheva, Israel; 2 Genome Diversity Center, Institute of Evolution, University of Haifa, 31905 Haifa, Israel

## Abstract

**Background:**

RNAmute is an interactive Java application which, given an RNA sequence, calculates the secondary structure of all single point mutations and organizes them into categories according to their similarity to the predicted structure of the wild type. The secondary structure predictions are performed using the Vienna RNA package. A more efficient implementation of RNAmute is needed, however, to extend from the case of single point mutations to the general case of multiple point mutations, which may often be desired for computational predictions alongside mutagenesis experiments. But analyzing multiple point mutations, a process that requires traversing all possible mutations, becomes highly expensive since the running time is *O*(*n*^*m*^) for a sequence of length n with m-point mutations. Using Vienna's RNAsubopt, we present a method that selects only those mutations, based on stability considerations, which are likely to be conformational rearranging. The approach is best examined using the dot plot representation for RNA secondary structure.

**Results:**

Using RNAsubopt, the suboptimal solutions for a given wild-type sequence are calculated once. Then, specific mutations are selected that are most likely to cause a conformational rearrangement. For an RNA sequence of about 100 nts and 3-point mutations (*n *= 100, *m *= 3), for example, the proposed method reduces the running time from several hours or even days to several minutes, thus enabling the practical application of RNAmute to the analysis of multiple-point mutations.

**Conclusion:**

A highly efficient addition to RNAmute that is as user friendly as the original application but that facilitates the practical analysis of multiple-point mutations is presented. Such an extension can now be exploited prior to site-directed mutagenesis experiments by virologists, for example, who investigate the change of function in an RNA virus via mutations that disrupt important motifs in its secondary structure. A complete explanation of the application, called MultiRNAmute, is available at [1].

## Background

### Introduction

The secondary structure of an RNA molecule is a representation of the pattern, given an initial RNA sequence, of complementary base-pairings that are formed between the nucleic-acids. The sequence, represented as a string of four letters, is a single strand consisting of the nucleotides A, C, G, and U, which are generally assumed to pair to form a secondary structure with minimum free energy. As such, the secondary structure of RNA is experimentally accessible based on minimum free energy calculations, thus making its computational prediction a challenging but practical problem: it can be directly tested in the laboratory with minimal experimental effort relative to, for example, RNA tertiary structure. Moreover, there is a well known correspondence between the secondary structure of RNA and the molecule's ultimate function.

RNA viruses are known to possess unique secondary structures. The secondary structure of an RNA virus such as the Hepatitis C Virus (HCV) is mostly elongated due to the large number of base pairings that are formed, thereby lowering its free energy considerably and making the virus much more thermodynamically stable than a random RNA sequence. The typical stem-loop structure motif of an RNA virus, which consists of a long stem (a chain of base pairs) that ends in an unpaired loop, has been experimentally observed to play a significant role in both virus replication and translation initiation. For example, in HCV, disruptive mutations were found to cause a structural change that directly led to either an alteration in virus replication [2,3] or to a dramatic reduction in translation initiation [4].

### RNA folding prediction

The folding prediction problem of the secondary structure of RNAs has been an area of active research since the late 70's. Dynamic programming methods were developed in [5] and in [6,7] for computing the maximum number of base pairings in an RNA sequence. Energy-minimization methods by dynamic programming [8,9] have led to Zuker's Mfold prediction server [10] and the Vienna RNA package [11,12]. The predictive accuracy of these packages was improved by incorporating expanded energy rules [13], derived from an independent set of experiments, into the folding prediction algorithm.

### RNA mutation prediction

The folding prediction problem described above is the most fundamental problem in RNA bioinformatics. The related RNA mutation prediction problem, in turn, is a sub-problem that uses the methods developed for RNA folding prediction multiple times to predict various mutation combinations. From a computer program's perspective, mutation prediction can be viewed as an outer loop that uses RNA folding prediction in its inner loop. The mutation prediction problem, however, presents several computationally challenging issues, mainly in the generalization to multiple-point mutations, which can become computationally heavy if a 'brute-force' strategy of calculating all possible mutations is used without devising a unique approach. We propose to solve this problem by using suboptimal folding prediction solutions, described in the next section, which offer a practical method for achieving high computational efficiency.

The mutation prediction problem was initially investigated in [14,15] and has been revived in [16,17]. The first publicly available computerized tools for RNA mutation prediction, which only account for single-point mutation predictions, are the Java tool called RNAmute [18] and a web server called RDMAS [19]. Neither of the tools can handle multiple-point mutations, though the authors in this work have already extended RNAmute to calculate all multiple-point mutations in a 'brute-force' manner (unpublished), revealing that this is a computationally heavy task.

### RNAmute: RNA secondary structure single-point mutation analysis tool

RNAmute is an interactive Java tool that, given an RNA sequence, calculates the secondary structure of all single point mutations and organizes them into categories according to their distances from the predicted wild-type structure. More details are available in [18]. For grouping and analyzing the point mutations, RNAmute utilizes mathematical theorems that relate to eigen-decomposition of the Laplacian matrix [20,21] corresponding to Shapiro's coarse-grain tree graphs [14]. The Vienna RNA package [11] is currently used as the core for RNAmute and for the RDMAS server [19] that also analyzes point mutations. Future extensions may include RNAshapes [22] for the coarse-grain representation and the RNAforester [23] for tree comparisons.

### Motivation for an efficient extension to analyze multiple-point mutations

The examination of the phenotypic data based on Hepatitis C Virus (HCV) experiments [2,3] presents a typical example of the powerful potential of RNAmute. In the structural analysis of 5BSL3.2 via mutagenesis experiments, RNAmute could have assisted as a pre-processing step, performed before the site-directed mutagenesis experiments, to provide a selection of mutations superior to that achieved by trial and error. RNAmute could have identified locations likely to disrupt certain motifs in the RNA secondary structure that are known to be functionally important for virus replication. Another example that highlights the application potential of RNAmute is the structure of the stem-loop IIIc of the Hepatitis C Virus 1b 5' untranslated region (5' UTR) [4]. In this case, RNAmute can predict a single point mutation that causes a dramatic reduction in the translation initiation of the virus.

Thus, extensions of RNAmute should enable preliminary analyses before deciding which mutations to employ experimentally for the disruption of certain important motifs in the RNA secondary structure. Our innovative approach presents an automated technique for considering some of the sophisticated secondary structure scenarios beyond a local disruption or formation of a single Watson-Crick base pairing, which proved misleading in the past, as was the case in [4] that was discussed in [18]. Although this specific case could have been resolved using the 'primitive' RNAmute described in [18] for predicting a single point mutation that hinders the translation initiation, more advanced cases will need an efficiently running, multiple-point mutation, 'extended' RNAmute, as proposed here.

Using RNAsubopt [24], our method extends the applicability of RNAmute to multiple-point mutations. In general, the same type of analysis could be done using the suboptimal solutions obtained by Mfold [10,25]. However, for practical reasons concerning our specific application of multiple-point mutations we utilized the Vienna RNA package since Mfold uses pre-defined filters for filtering suboptimal structures. In developing our method, the motivation was to start with all suboptimal solutions as provided in Vienna's RNAsubopt [24] and to experiment with various filters of our own, comparing our application's performance to that of the original RNAmute [18] with Vienna's RNAfold. In the future, we plan to incorporate Mfold into RNAmute alongside Vienna's RNAfold and RNAsubopt. Note that both RNAsubopt and RNAfold are used with the "no lonely pair" option to conform with the Vienna web server.

The original RNAmute [18] analyzes only one-point mutations for a given RNA sequence. Although a single point mutation may sometimes cause a secondary structure rearrangement of an RNA molecule, often it is essential to introduce more than a single point mutation to alter the RNA secondary structure. The second version of RNAmute (in progress) that followed the original [18] and was implemented before the work described here is already capable of dealing with multiple point mutations by trying all possible m-point mutations. However, if the length of the sequence is n and the number of point mutations is m, the running time for the number of point mutations tried will be *O*(*n*^*m*^), which, computationally, is highly expensive. To estimate the run time, analysis of a sequence length of about 100 nts with 3-point mutations would require at least several hours on a typical PC. To overcome this problem, we present a faster method in which we do not need to simulate all possible m-point mutations in the sequence; instead, after executing RNAsubopt [24] once on the wild-type sequence, we find them directly from the suboptimal solutions provided by the Vienna RNA package.

## Results

### Algorithm

**Figure 1:**
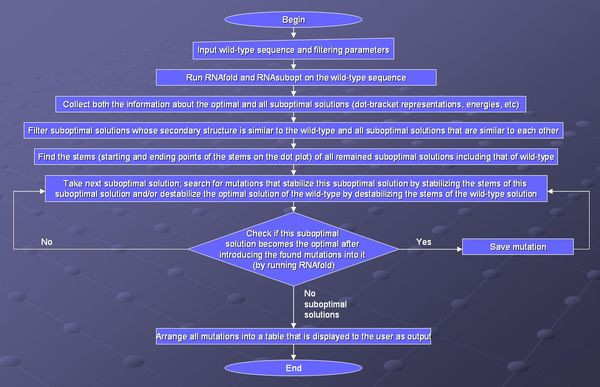
**Flowchart of the Proposed Procedure**. A summary of each step in the suggested procedure.

The algorithm consists of several steps performed consecutively. First, given a wild-type sequence with several input parameters, the suboptimal solutions of the minimum free energy folding prediction are calculated. This step is followed by a suboptimal solutions filtering step. Next, the beginning and end points of each stem in the suboptimal solution results are calculated. Finally, m-point mutations that disrupt the optimal solution are calculated. A summary of all the steps in the procedure is given in the flowchart available in Figure 1. Each step is described in detail below.

### Calculating suboptimal solutions

After running the program, it calculates the dot-bracket representation of the optimal secondary structure of the given sequence, using the RNAfold routine of the Vienna RNA package, and the dot-bracket representations of all suboptimal secondary structures that are obtained using the RNAsubopt routine of the Vienna RNA package with some parameter -e (for calculating suboptimal structures within a range of kcals/mol of the mfe) are also calculated. This parameter is chosen by the user. The lower limit of e is 0. Regarding the upper limit, as will be elaborated in the Discussion Section, it is recommended to start for example from e = 15 for short sequences of about 70 nts with 3-point mutations and then to perform consecutive trials with increasing e until the optimal value is found for a particular case. In the case of sequences of about 70 nts and 3-point mutations, increasing e to high values such as 30 instead of 15 will yield a running time of days instead of seconds/minutes and is not desired. The program saves the optimal structure as **"Opt" **and all the suboptimal structures as a list called **"Subopts"**.

### Filtering suboptimal solutions

We use three filters on the "Subopts" list:

1) The first filter removes all suboptimal solutions for which the distance of their dot bracket representations from "Opt" is less than a parameter value ("dist1") as specified by the user ("dist1" is in the range from 0 to n). The distances are computed as described below.

2) The second filter is designed to simply discard the suboptimal solutions that most likely will not become the optimal solution after the introduction of an m-point mutation. For example, if the dot bracket representation of the optimal solution of the sequence **UGCCUGCCUCUUGGGAGGGGC **is **.(((..((((....))))))) **and the dot bracket representation of one of the suboptimals is **..((........)).......**, then it is clear that no one-point mutation can cause the optimal to become suboptimal. Compared to the other filters, the effect of such a filter is minor, and indeed it can be easily shut off as explained in connection with a threshold parameter called DIFF that will be described below. To generate such a filter, one could consider scenarios of hypothetical mutations in which a previously suboptimal solution becomes the optimal, but in such scenarios the location of the mutation is unknown, and therefore, we cannot measure the stability of such a structure using the Zuker-Turner energy rules [13]. However, we can apply a simplistic, highly approximate model that suits our requirements, one that will calculate the relative stabilities of the secondary structures of the optimal and all suboptimal solutions using a weighted Nussinov model [7] for assessing the strength of the base pairings. The base pairs CG and GC that are composed of three hydrogen bonds are given a score of 3, base pairs AU and UA that are composed of two hydrogen bonds are given a score of 2, and base pairs GU and UG that are traditionally considered a weaker bond compared to the former (e.g., early estimations described in [26]) are given a score of 1. After this calculation the filter removes those suboptimal solutions with relative stabilities that are lower than that of the optimal as a consequence of the introduction of "numMuts" mutations (hypothetical mutations for which their exact location is unknown) into the wild-type sequence. For more information on how this filter operates and how to shut it off, see 'Additional file 1:Filter2' for supplementary information on the second filter.

3) The third filter removes the suboptimal solutions that are closest to each other, i.e., if the distance between two suboptimal solutions is less than a parameter "dist2" that is specified by the user ("dist2" is in the range from 0 to n), then one of the suboptimal solutions will be removed. As a pre-processing step, we prefer to remove solutions whose distances from the optimal are smallest and those deemed as less stable solutions. For this reason we sort all the remaining (after the two filters above) suboptimal solutions according to their distance from optimal in descending order and subsequently sort them according to their energy calculated by RNAsubopt. Only after the program is done with both sorting tasks is the third filter applied. We start from the first suboptimal solution (with the largest distance from optimal and the most stable), and check the distance of this solution against all other solutions. Each of the following solutions for which the distance from the first solution is less than "dist2" will be removed from the list, but the first solution remains in the list. After reaching the end of the list, the second suboptimal solution becomes the first, and so on.

If the chosen parameters dist1 and dist2 (both ranging from 0 to n) are large numbers relative to the length of the sequence, then filtering is a fast process because most suboptimal solutions were filtered already with the first filter. Thus, the third filter, which has a quadratic running time of O(subs^2^) because we compare all pairs of suboptimal solutions, also appears to be fast. The running time of the first filter is O(subs), where "subs" is the number of suboptimal solutions obtained by running RNAsubopt on the wild-type RNA sequence.

### Calculating the distance between two dot-bracket representations

In the future, we will offer a choice between three methods for calculating the distance between two dot-bracket representations of RNA secondary structures, but here we only used the two computationally faster techniques to present the methodology. The first method is Vienna's RNAdistance, which calculates a tree-edit distance by default. The second, more approximate method was developed to save time because we had too many suboptimal solutions to compare. Using this method we simply run the two dot-bracket representations in parallel and calculate the number of mismatches, or, equivalently, the Hamming distance. The running time is O(n), where n is the length of the dot-bracket representation. Finally, distances can also be calculated using the base pair distance that has been used in many previous studies (also available as an option in Vienna's RNAdistance). Similar to the Hamming distance, the base pair distance can be calculated efficiently with a running time of O(n). The current version of our application includes both the Hamming distance and the base pair distance as options for the user; the more expensive tree-edit distance could be added in the future. For example, suppose we have the following two dot brackets:

((((.....))))

.((((....))))

The Hamming distance between the two dot-bracket representations is 2 (which is, in this case, the same value as the tree-edit distance calculated using RNAdistance with its default parameters), whereas the base pair distance is 8.

### Calculating the stems

After we filter the suboptimal solutions, we calculate the stems for each suboptimal solution by calculating the starting and ending points of each stem in the suboptimal solution.

For example, one of the suboptimal solutions for the given sequence **UGCCUGCCUCUUGGGAGGGGC **is:

..(((.((....)).)))...

**Figure 2:**
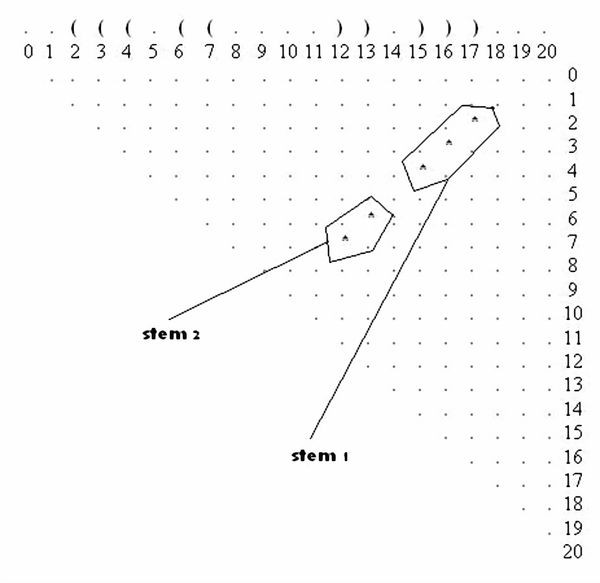
**Suboptimal Solution in a Dot Plot**. Illustration of a suboptimal solution example in a dot plot. The solution is obtained by running Vienna's RNAsubopt for the sequence **UGCCUGCCUCUUGGGAGGGGC**.

The dot plot (an *n *× *n *matrix with dots in the cells that correspond to base pairs) for this suboptimal solution has two stems (Figure 2). In all the dot plots presented in this work, it should be noted that we start the numbering from zero, and when referring to sequence positions in the dot plots this should be taken into account. The stems are represented by the starting and ending points, so the start of the first stem on this plot is location (2, 17) and the end of the first stem is location (4, 15). Similarly, the starting and the ending points of the second stem are (6, 13) and (7, 12) respectively.

### Calculating m-point mutations that disrupt the optimal solution

We begin by searching for locations at which mutations in the dot plot may: (1) stabilize the suboptimal solution; (2) destabilize the optimal solution; and (3) simultaneously stabilize the suboptimal solution and destabilize the optimal solution. The stabilizing mutations in our case are mutations that extend the existing stems, or those that introduce an additional stem (with length > 1) near an existing stem, without disrupting any base pairs in the existing stems. The destabilizing mutations in our case are mutations that disrupt some existing base pairs in the optimal solution without disrupting any base pairs in the suboptimal solution.

**Figure 3:**
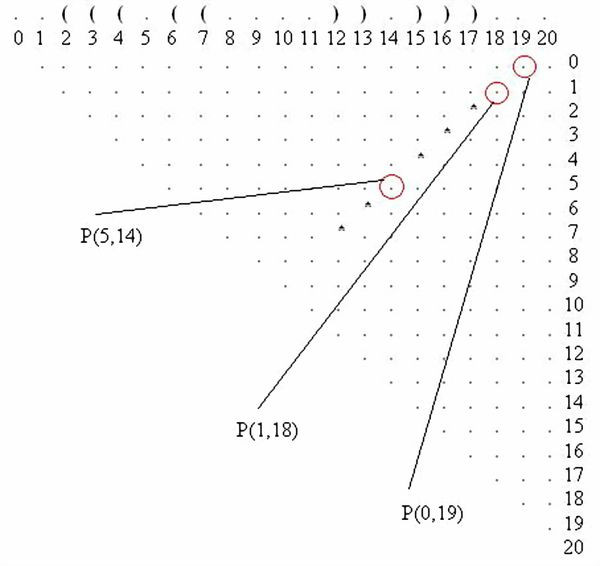
**Stabilizing Mutations in the Dot Plot**. The stabilizing mutations found by applying the proposed method on the dot plot in Figure 1. The stabilizing mutations are highlighted in circles.

For example, location P(5, 14) for a mutation in the dot plot of Figure 2 signifies that a mutation in either nucleotide 5 or 14 on the RNA sequence forms a base pair between nucleotides 5 and 14. Note that P(5, 14) extends both stem1 and stem2, even connecting them, and as such it stabilizes the suboptimal solution shown in Figure 2. Additionally, P(1, 18) and the double location P(1, 18), P(0, 19) are also stabilizing locations because they extend stem1. All the "stabilizing" mutations that we found on the dot plot (Figure 2) are highlighted by circles in Figure 3.

Therefore, if we are only searching for stabilizing single point mutations, then P(5,14) or P(1,18) are candidates but not P(0,19) as it forms a lonely base pair, which is not stable, and as such it should be discarded. In the case of stabilizing two-point mutations there are also two possibilities, P(5,14), P(1,18) and P(1,18), P(0,19), while a three-point mutation would incorporate P(5,14), P(1,18), P(0,19). There are no four or greater-than-four point mutations for this suboptimal solution if we are considering only stabilizing mutations. The locations P(8,11) and P(9,10) are not stabilizing locations because they will lower the hairpin loop to fewer than three nucleotides, which is unstable. On the other hand, it is possible that mutations in the hairpin are good if these mutations destabilize the optimal solution.

**Figure 4:**
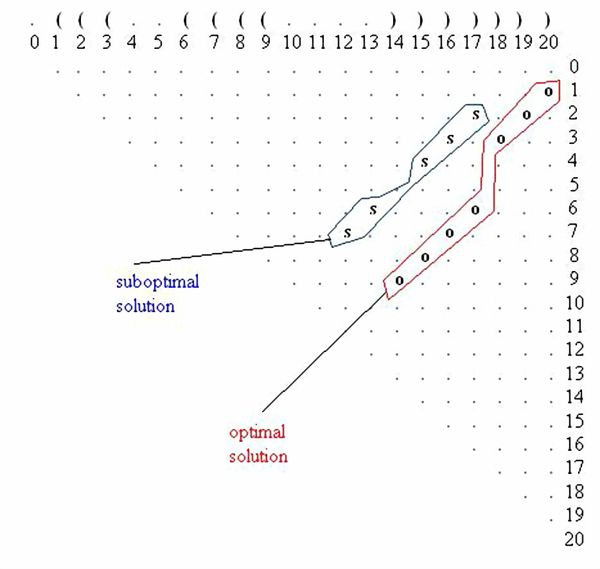
**Optimal and Suboptimal Solutions in a Dot Plot**. Both optimal and suboptimal solutions in a dot plot, drawn for the case of Figure 1, running Vienna's RNAsubopt for the sequence **UGCCUGCCUCUUGGGAGGGGC**.

**Figure 5:**
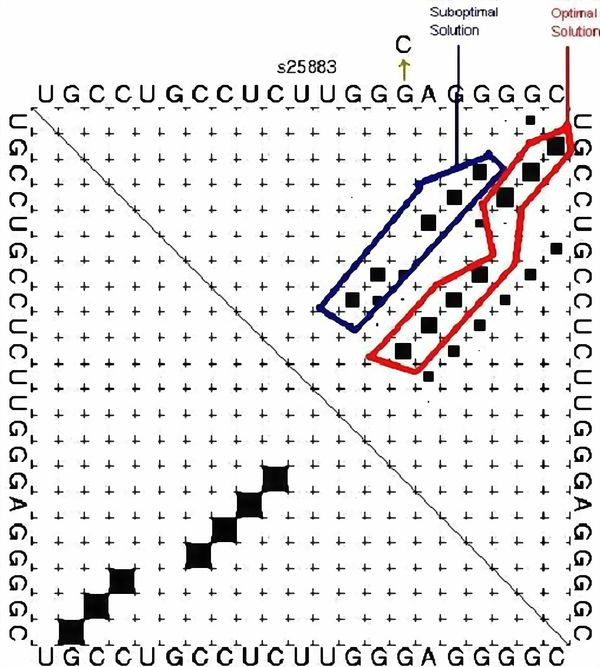
**Optimal and Suboptimal Solutions in a Probability Dot Plot**. The full probability dot plot, drawn for the case of Figure 1, as a result of running Vienna's RNAsubopt for the sequence **UGCCUGCCUCUUGGGAGGGGC**.

**Figure 6:**
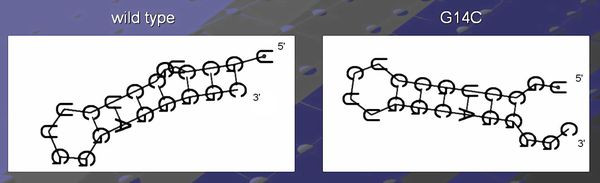
**Secondary Structure Drawings for the Wild-type and Mutant**. Secondary structure drawings for the wild-type and the mutant as a consequence of applying the rearranging point mutation found by our method, for the example in Figure 1 (the example is for the sequence **UGCCUGCCUCUUGGGAGGGGC**).

Using the same sequence (**UGCCUGCCUCUUGGGAGGGGC**), the optimal solution is **.(((..((((....)))))))**, and the corresponding dot plot is shown in Figure 4. In this Figure the suboptimal solution that appears in Figures 2 &3 and an optimal solution for the RNA sequence are observable. Figure 5 shows the probability dot plot obtained by running the Vienna RNA package on the same sequence. Based on Figures 4 &5, we can conclude that mutation G14C in location P(5,14) stabilizes the suboptimal solution by forming a CG base pair between nucleotides 5 and 14. This same mutation, however, also destabilizes the optimal solution by breaking a GC base pair between nucleotides 9 and 14. Therefore, the mutation G14C is both stabilizing and destabilizing. On the other hand, mutation G5C at the same location P(5,14) is only a stabilizing mutation, because it also forms a base pair between nucleotides 5 and 14 in the suboptimal solution and connects "stem 1" and "stem 2", but it has no disruptive effect on the optimal solution. Each of these mutations is worth checking, but we may assume that mutation G14C will have a stronger effect on the conformational rearrangement of the optimal solution. And indeed, if we introduce mutation G14C and use the Vienna RNA package, we can confirm that this is a conformational rearranging mutation (Figure 6). A rearranging mutation means a drastic change in one of the secondary structure motifs as inspected by eye, such as two new hairpins forming instead of one, etc.

Mutations G18C and G19A at locations P(1,18) and P(0,19) (Figure 3) are also both stabilizing and destabilizing mutations, while mutations on the hairpin of the suboptimal solution, at locations P(8,11) and P(9,10), are only destabilizing mutations. For example, mutation U8G at P(8,11) disrupts a UA base pair between bases 8 and 15 in the optimal solution, but this mutation has no effect on base pairs in the suboptimal solution.

### Implementation

First, we describe the optional modes of operation available to the user according to the problem at hand ("method") when implementing the proposed methodology. Second, in Testing we analyze the results of two artificial examples in detail, reporting running times, parameter usage, and possible limitations. Third, we show two practical implementation examples taken from the full P5abc subdomain of the *Tetrahymena thermophila *group I intron ribozyme and the 5BSL3.2 sequence of a subgenomic hepatitis C virus (HCV) replicon.

After identifying the stabilizing and destabilizing locations, the program calculates m-point mutations using these detected locations. There are four options, depending on the desired running time vs. the number of mutations to be tried, for calculating mutations:

1) In the first option, we only take into account the stabilizing locations and we can only extend the existing stems without making any new stems. The number of mutations in this option is bounded by (2*s*)^m ^* 2^*m*^, where s is the number of stems in the suboptimal solution and m is the number of point mutations. The expression (2*s*) is needed because mutations may be introduced at both ends of the stem, and 2^*m *^is included because in each detected location we may introduce two different mutations. The number of mutations will be much lower because in practice the stems are relatively close to each other and it is impossible to perform m mutations near each stem; and in some cases, it will be impossible to perform even a single mutation near the ends of most stems. Even for the worst cases, however, the running time is better than in the existing version of RNAmute when (3*n*)*^m^*mutations are tried, *n *being the length of the sequence and *s *<<*n*. Of course, the running time also depends on the number of suboptimal solutions found, but if we take only different suboptimal solutions and use large values for dist1 and dist2, we will obtain only about 5–20 different suboptimal solutions.

2) In the second option, we take into account not only mutations that extend existing stems, but also those that create a new stem of length >1 between existing stems. The running time of this option is greater than that for the first option but still better than that of RNAmute. As in the first option, in most cases this option is also fast. But because extending the existing stems is more stable than forming a new short stem near the existing one, in most cases it is enough to use the first option instead of the second option.

3) Similar to the first option, this option also considers destabilizing mutations. In practice, the number of destabilizing mutations is usually relatively low, and therefore, the running time of this option is affordable for more than 4–5 point mutations. But in some cases where the number of possible destabilizing mutations is large, the running time is close to that of RNAmute.

4) A combination of options 2 and 3 that is similar to the second option, it also accounts for destabilizing mutations. For the same reason stated above in the second option, it is often enough to ignore option 2, thereby choosing option 3 over option 4.

For each m-point mutation found, we check the dot-bracket distance of the secondary structure of the mutated sequence, after applying RNAfold on it, against the optimal secondary structure of the wild-type sequence. If the distance is greater than or equal to dist1, we print this mutation.

### Input

Input to our program includes:

1. Query RNA sequence – **"seq"**.

2. Energy parameter for RNAsubopt routine **"e" **(for advice on how to choose its values based on sequence length, see the Discussion Section).

3. Distance **"dist1" **for filtering the suboptimal solutions that are close to the optimal. Note: it is recommended that the value for dist1 be about 25% of the sequence length, and this value should be lowered if more solutions are desired.

4. Distance **"dist2" **for filtering the suboptimal solutions that are close to each other. Same advice above on how to pick a value for dist1 holds for dist2.

5. **"numMuts" **– number of point mutations that should be introduced into the RNA sequence.

6. **"method" **– type of algorithm that will be used to calculate the final m-point mutations.

The recommended parameter values are found in the Discussion Section.

### Testing

We test our method by demonstrating it on two examples using artificially generated sequences. The results are described in detail. For our first example, we executed our procedure on the following sequence of length 23 nts:

CCUUAACCAGCAAAAACUGCUGG

**Figure 7:**
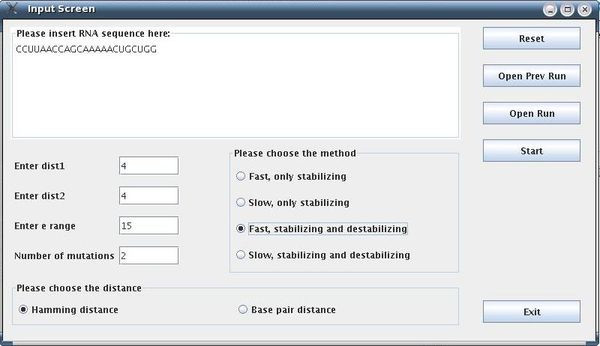
**Input Screen of Artificial Example I**. Input screen of our procedure for an artificial example (the example is for the sequence CCUUAACCAGCAAAAACUGCUGG).

**Figure 8:**
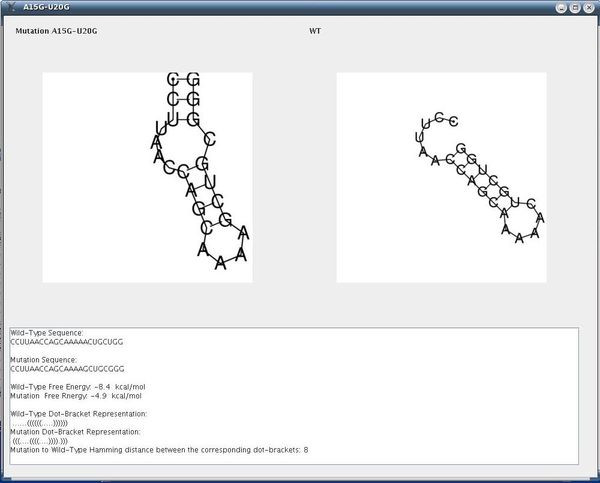
**Output Screen of a Rearranging Mutation in Artificial Example I**. Output screen of our procedure for the rearranging mutation A15G-U20G with the secondary structure drawings for the wild-type and the mutant, including additional measures (the example is for the sequence **CCUUAACCAGCAAAAACUGCUGG**).

**Figure 9:**
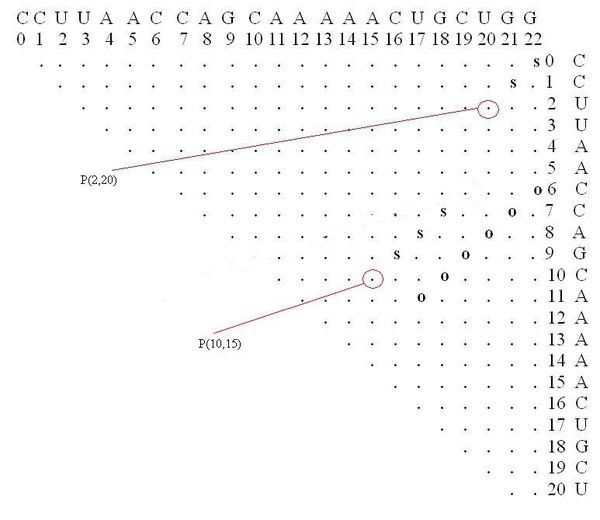
**Dot Plot of a Rearranging Mutation in Artificial Example I**. The dot plot corresponding to the rearranging mutation A15G-U20G (the example is for the sequence **CCUUAACCAGCAAAAACUGCUGG**).

The following parameters were used: dist1 = 4, dist2 = 4, e = 15, numMuts = 2, method = 3, distance = Hamming. The input screen appears in Figure 7. The running time reported was 30 sec on a typical PC. About 500 rearranging mutations were found (out of a possible total for this example of about 2300). Fewer mutations can be obtained by decreasing the value for e or increasing the values of dist1 and dist2. For example, when dist1 is larger, out of the 500 rearranging mutations originally detected, only those exhibiting a greater likelihood to be rearranging mutations are left; if dist2 is increased, fewer mutations that degenerate to similar secondary structures are left. One of the rearranging 2-point mutations (A15G-U20G) is reported in Figure 8 and its corresponding dot plot in Figure 9. Examining the dot plot reveals the optimal (denoted by 'o') and suboptimal (denoted by 's') solutions from which we obtained the structure in Figure 8 after mutations in places P(2,20) and P(10,15). The mutation A15G stabilizes the suboptimal solution by elongating the stem (from 3 to 4), and the mutation U20G is both stabilizing (it elongates the stem in the suboptimal solution from length 2 to length 3) and destabilizing (it breaks a base pair 20U-8A in the optimal solution).

**Figure 10:**
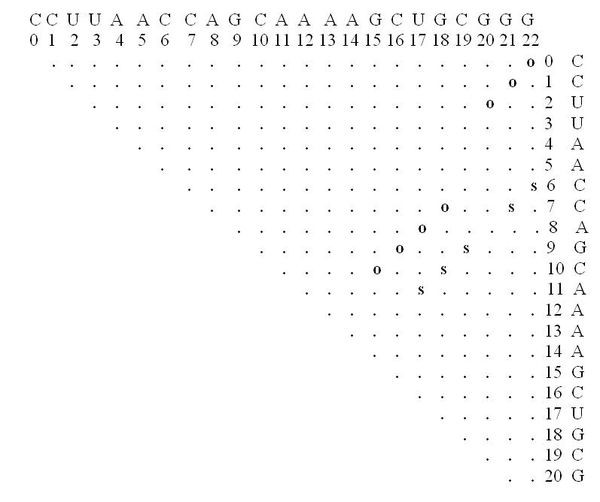
**Dot Plot with a Suboptimal Structure for Artificial Example I**. The dot plot for a sample suboptimal solution, and the optimal solution, corresponding to the rearranging mutation A15G-U20G (the example is for the sequence **CCUUAACCAGCAAAAACUGCUGG**).

After undergoing mutation, the suboptimal structure becomes the optimal and vice versa (Figure 10). Some of the other mutations at positions P(2,20) and P(10,15) that produce the same secondary structure are C10U-U20A, A15G-U20A. Note that C10U is both a stabilizing and destabilizing mutation (see Figure 9), as well as U20A.

For comparison, we ran RNAmute with multiple-point mutations on the same sequence and found that about 20% of all possible 2-point mutations are relatively rearranging mutations (relative rearranging means that the distance from the wild-type differs by 25% from the size of the sequence, not necessarily a drastic change to one of the secondary structure motifs that one can inspect by eye from the secondary structure drawings). Our program succeeded in finding almost all of these 2-point mutations (about 99%) because we used small distance values while the value of e was even larger than the energy of the optimal structure. Using RNAmute with multiple-point mutations is computationally very slow, and with it we are almost unable to analyze sequences longer than in the example above and/or those with more than 2-point mutations. This shows the necessity for our efficient procedure.

For longer sequences and for those with more mutations, the number of rearranging mutations is very large. A trial run of RNAmute on sequences of about 50 nt with 2-point mutation showed that more that 10% of all the m-point mutations were rearranging. If we use a smaller value for e and a larger value for dist2, however, we obtain fewer rearranging mutations. Therefore, if we want the program to terminate fast, we will lose a significant number of desired mutations. Consequently, to detect more rearranging mutations requires a larger value for e and a smaller value for dist2.

In terms of this example, the following scenarios may occur. There may exist a highly unstable suboptimal solution which, after receiving an m-point mutation, becomes highly stable, thus becoming the new optimal solution. Finding such mutations requires that the value of e be increased. In addition, there may be two similar secondary structures, but only one of these structures will become the optimal solution after the insertion of mutations into both structures. If we filter the first structure, for example, with the third filter, we remain with the second one, and as such we may also miss some desired mutations. To overcome this problem, a smaller value of dist2 should be tried, but in so doing the running time will increase.

Admittedly, what cannot be overcome are the cases in which mutations destabilize both the optimal and the suboptimal solutions. Such mutations may destabilize the optimal solution more than the suboptimal one, and the suboptimal may, as a consequence, become the optimal solution. Checking such mutations will require running times no shorter than those of RNAmute with multiple-point mutations. It is also possible that mutations (especially if the number of mutations is larger than 4–5) form some stems that do not appear in both the optimal and the suboptimal solutions; our method cannot find such mutations, the number of which is relatively small. For example, in the sequences we checked when comparing the performance of RNAmute with multiple-point mutations, undetectable mutations comprised no more than 5% of all the rearranging mutations. This number will increase, however, if we use more than 4–5 point mutations.

For the second example, we ran the program on the following sequence comprising 93 nts:

CCGGAAGAGGGGGACAACCCGGGGAAACUCGGGCUAAUCCCCCAUGUGGACCCGCCCCUUGGGGUGUGUCCAAAGGGCUUUGCCCGCUUCCGG

using the parameters dist1 = 25, dist2 = 25, e = 15, numMuts = 3, method = 3, and distance = Hamming.

**Figure 11:**
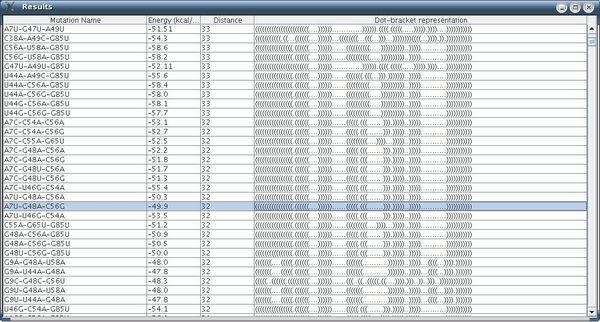
**Mutation Group List Screen in Artificial Example II**. Mutation group list screen as a result of our procedure, for the case of 3-point mutations (the example is for the sequence **CCGGAAGAGGGGGACAACCCGGGGAAACUCGGGCUAAUCCCCCAUGUGGACCCGCCCCUUGGGGUGUGUCCAAAGGGCUUUGCCCGCUUCCGG**).

**Figure 12:**
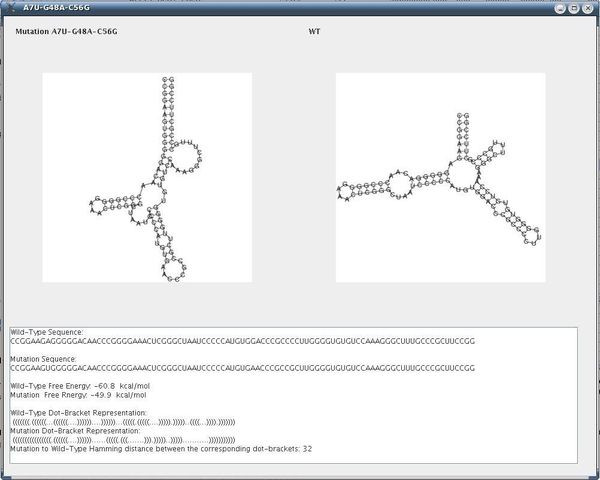
**Output Screen of a Rearranging Mutation in Artificial Example II**. Output screen of our procedure for a rearranging 3-point mutation with the secondary structure drawings for the wild-type and the mutant, including additional measures (the example is for the sequence **CCGGAAGAGGGGGACAACCCGGGGAAACUCGGGCUAAUCCCCCAUGUGGACCCGCCCCUUGGGGUGUGUCCAAAGGGCUUUGCCCGCUUCCGG**).

The running time reported in this example for our procedure was 7 minutes, and the number of rearranging mutations found was about 2000. An example of a rearranging 3-point mutation can be observed in Figure 11 (selected among a list of mutations) while Figure 12 contains more information about the same mutation.

Table 1 contains benchmark times, both for the examples reported above and for sample sequences of length n = 20, n = 40, n = 60, n = 80, and n = 120 nts respectively. For these sample sequences, all tests were performed using the parameters: method = 3, distance = Hamming, and dist1 and dist2 were taken to be 25% of the length n of the sequence (as recommended in the Input subsection for an initial run). The base sequence used for these tests was the following 20 nts long sequence:

**Table 1:** Benchmark Times for the Proposed Procedure.

A. Generated test cases					
*dist1*	dist2	n	e	numMuts	Time (sec)

5	5	20	8	**1**	**1**
5	5	20	8	**2**	**5**
5	5	20	8	**3**	**21**
5	5	20	8	**4**	**63**
5	5	20	8	**5**	**124**
10	10	40	8	**1**	**3**
10	10	40	8	**2**	**14**
10	10	40	8	**3**	**64**
10	10	40	8	**4**	**341**
**10**	**10**	**40**	8	**5**	**1448**
**15**	**15**	**60**	8	5	**23469**
10	10	40	**7**	2	**11**
10	10	40	**8**	2	**14**
10	10	40	**9**	2	**25**
10	10	40	**10**	2	**43**
10	10	40	**12**	2	**103**
10	10	40	**14**	2	**328**
**5**	**5**	**20**	8	2	**5**
**10**	**10**	**40**	8	2	**14**
**15**	**15**	**60**	8	2	**35**
**20**	**20**	**80**	8	2	**135**
**30**	**30**	**120**	8	2	**163**
**5**	**5**	**20**	8	3	**21**
**10**	**10**	**40**	8	3	**64**
**15**	**15**	**60**	8	3	**266**
**20**	**20**	**80**	8	3	**512**
**25**	**25**	**100**	8	3	**1032**
**30**	**30**	**120**	8	3	**1827**
**40**	**40**	**160**	8	3	**5176**

B. Examples reported in the Results Section:					

*dist1*	dist2	n	e	numMuts	Time (sec)

4	4	23	15	2	**23**
25	25	93	15	3	**437**
15	15	64	15	2	**172**
12	12	45	12	2	**158**

Benchmark times for some generated test cases and for the examples reported in the Results Section, performed on a stand-alone PC with a 2.8 GHz Intel dual-core processor. Highlighted are the parameter values that were varied each time.

UGCCUGCCUCUUGGGAGGGG

And this sequence was repeated (concatenated to itself) to form the longer sequences of length n = 40, n = 60, n = 80, and n = 120 nts reported in the table. The calculations were performed on a stand-alone PC with a 2.8 GHz Intel dual-core processor.

### Examples for a success of our procedure

**Figure 13:**
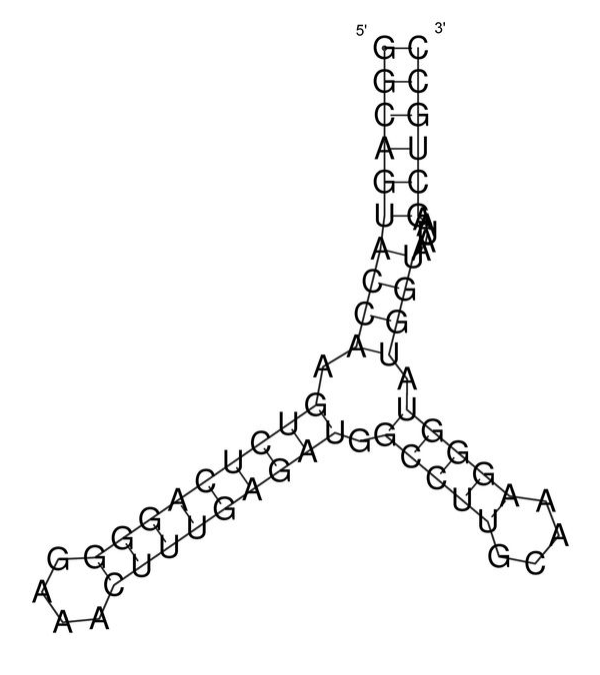
**Secondary Structure Drawn for the full P5abc Subdomain (wild-type)**. The secondary structure for the full P5abc subdomain of the *tetrahymena thermophila *group I intron ribozyme that appears in the NDB.

For the first example we used the full RNA sequence of the P5abc subdomain of the *Tetrahymena thermophila *group I intron ribozyme that appears in the Nucleic Acid Database (NDB) [27]. The purpose of this illustrative example on a well-known biological structure is to show that predictions of our suggested efficient procedure that take a few minutes coincide with predictions performed in a 'brute-force' manner by traversing all possible point mutations that take several hours. In the experiments of Wu and Tinoco [28], the authors initially took off 4 base pairs for convenience with their experiment, starting from the base pair 15 U – 30A and ending with the base pair 18G – 27 U of the structure drawn in Figure 13. In addition, the 19G – 26 U wobble base pair was replaced by a 19G – 26C Watson-Crick base pair and the 20G – 25C base pair was flipped to 20C – 25G. This truncated P5abc subdomain of [28] served as an illustrative example in [16] where it was analyzed with RNAmute using only single-point mutations, predicting that only an exceptional single-point mutation may lead to a conformational rearrangement. However, when inserting the full P5abc subdomain into RNAMute, it is computationally predicted that no single-point mutation will cause a conformational rearrangement. This illustrative example is small enough to try the 'brute-force' version of RNAmute for the case of two point mutations, traversing all two-point mutations in a calculation that takes several hours, finding that only a few two-point mutations located in the P5b stem-loop are predicted to cause a significant conformational rearrangement to a linear structure. We would like to find in a much more efficient manner, using our suggested procedure, those two-point mutations in the RNA sequence that will cause a conformational rearrangement to a linear structure of the full P5abc subdomain. Therefore, we execute our program on the sequence with the parameters dist1 = 15, dist2 = 15, e = 15, numMuts = 2, method = 3, and distance = Hamming.

The wild-type sequence of the full P5abc subdomain is:

GGCAGUACCAAGUCUCAGGGGAAACUUUGAGAUGGCCUUGCAAAGGGUAUGGUAAUAAGCUGCC

The dot bracket representation of the optimal secondary structure is:

((((((((((.(((((((((....))))))))).(((((....))))).)))).....))))))

The minimum free energy is -29.8 kcals/mole, and the predicted structure is shown in Figure 13.

After running our program on the wild-type sequence of the full P5abc subdomain, with a reported running time of 3 minutes, we obtained several suboptimal structures, in which one of them is a linear structure and its dot bracket representation is:

((((((((((..((((((((....((...))....)))))....)))..)))).....))))))

And its minimum free energy is: -15.9 kcals/mole.

**Figure 14:**
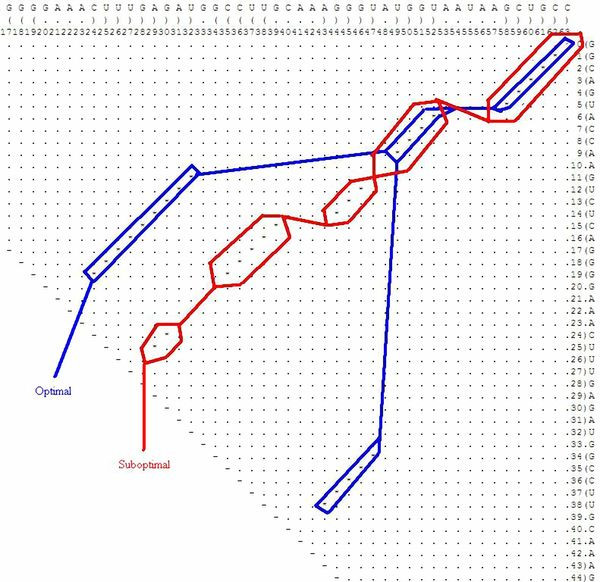
**Dot Plot with a Suboptimal Example for the full P5abc Subdomain**. The dot plot for a sample suboptimal solution, as well as the optimal solution, for illustrating the case of the full P5abc subdomain that appears in Figure 12.

This suboptimal secondary structure is clearly very unstable when compared to the optimal secondary structure. Figure 14 shows the dot plot with optimal and suboptimal secondary structures. The program finds several two-point mutations that cause the optimal solution to rearrange as the suboptimal solution becomes the optimal. These two-point mutations found in 3 minutes coincide with the ones found in a 'brute-force' manner that takes several hours.

**Figure 15:**
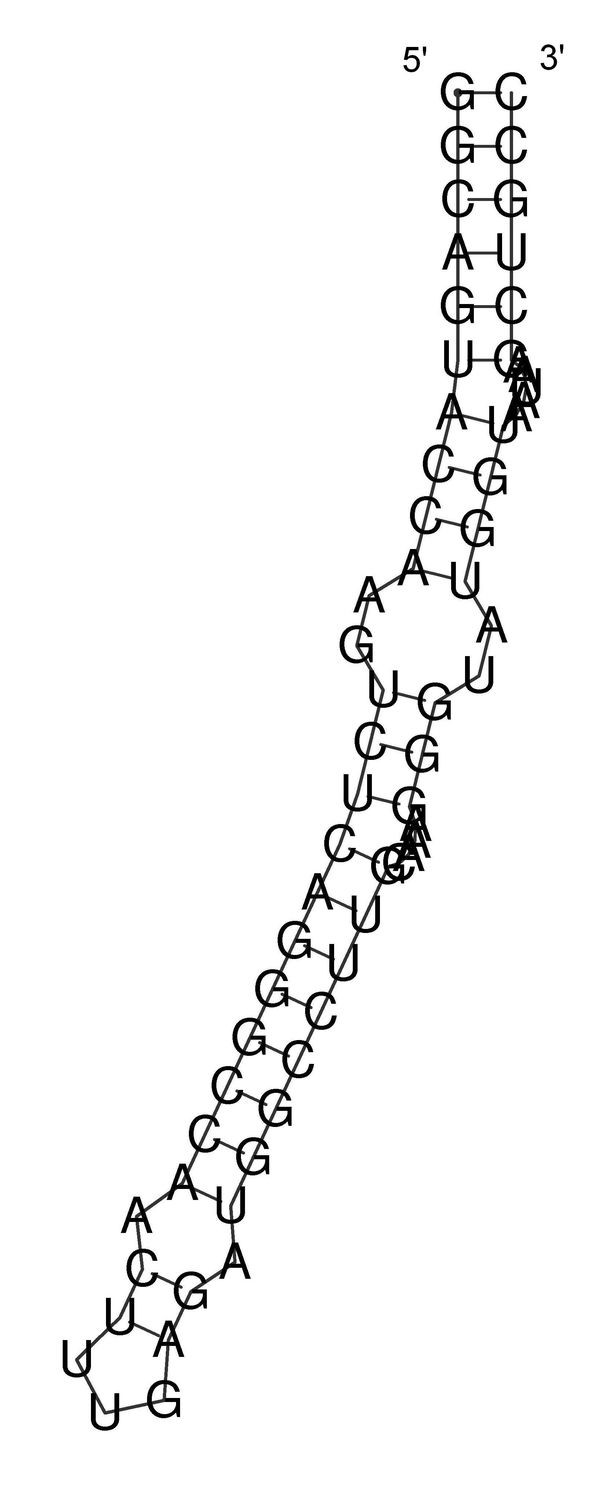
**Secondary Structure for the 2-pt mutation of the P5abc subdomain**. The secondary structure prediction for the mutant as a consequence of applying the 2-point mutation G20C-A21C on the full P5abc subdomain of Figure 12.

**Figure 16:**
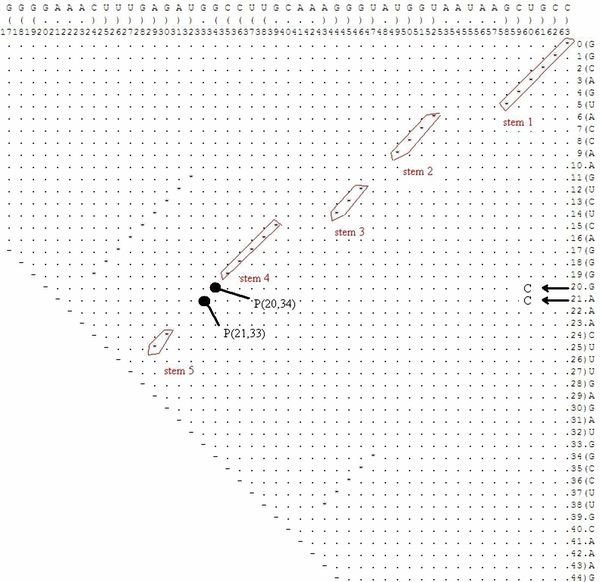
**Dot Plot with the Stabilizing Mutations for the full P5abc Subdomain**. The dot plot for the full P5abc subdomain in the case that appears in Figure 12, including the stems and the stabilizing point mutations that are highlighted.

For example:

1) The dot bracket representation of the secondary structure of the sequence with mutation G20C-A21C, using Vienna's RNAfold with the "no lonely pair" option is:

((((((((((..(((((((((((.((...)).))))))))....)))..)))).....))))))

a drawing of which appears in Figure 15. Inspecting the dot plot of Figure 16, note that both mutations G20C and A21C in locations P(20,34) and P(21,33) stabilize the suboptimal solution by extending "stem 4" (the base pairs between nucleotides 20 – 34 and 21 – 33 are formed), but these mutations have no effect on the base pairs of the optimal solution. In this case we obtain the suboptimal solution with three additional base pairs: two because of the mutations and a third one that was previously a lonely base pair.

2) Another case of three mutations G20C-G28C, G20C-G28U, G20C-G28A, in which their secondary structure has the following dot bracket representation using Vienna's RNAfold with the "no lonely pair" option:

((((((((((..(((((((((...((...))...))))))....)))..)))).....))))))

As in the previous case, mutation G20C is a stabilizing mutation, but the second mutation in position 28 of the sequence is a destabilizing mutation, which destabilizes the base pair 28G – 15C in the optimal secondary structure. In this case we obtain the suboptimal solution with one additional base pair.

For the second example, we take the 5BSL3.2 sequence of a subgenomic hepatitis C virus (HCV) replicon that was evaluated in [2] by site-directed mutagenesis experiments (see Figure 8 in [2], and "C84A/U86G disrupts the upper helix of 5BSL2.3" and "The upper helix is a scaffold" subsections in the aforementioned reference). This example shows the reproducibility of an example of published data for which introduced mutations in a mutagenesis experiment changed the structure and that our suggested computational procedure, given the original sequence, can successfully predict these mutations. The wild-type sequence of the 5BSL3.2 is:

AGCGGGGGAGACAUAUAUCACAGCCUGUCUCGUGCCCGACCCCGCU

**Figure 17:**
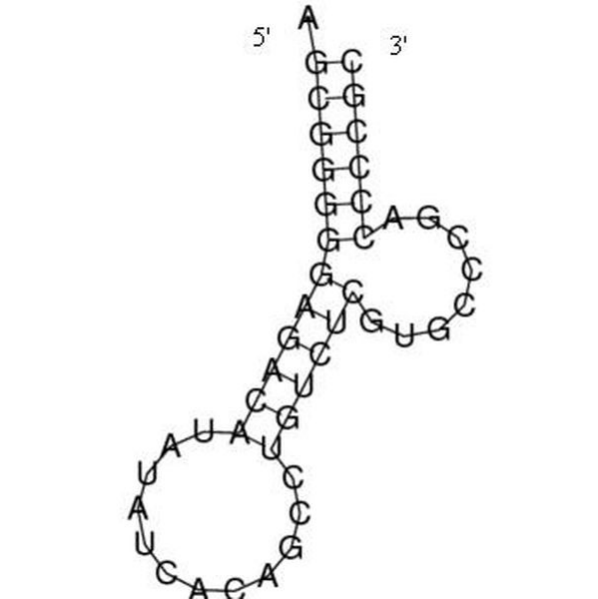
**Secondary Structure Drawn for the 5BSL3.2 (wild-type)**. The secondary structure for the 5BSL3.2 of a subgenomic hepatitis C virus (HCV) replicon, reported in Reference [2].

The wild-type is well-predicted by RNAfold, as can be observed in Figure 17. In [2], denaturating gels were extracted for the 5BSL3.2 wildtype, and for the two-point mutations C30A-U32G (corresponding to C84A-U86G in Figure 8 of [2]) and C30A-U32A. The gels indicated that in mutant C30A-U32G, which was find not viable, elimination of the C-G base pair by a change from C to A in position 30 and the change of the loop nucleotide 32 from U to G caused misfolding and disrupted the upper helix. Interestingly, mutant C30A-U32A was viable and maintained a similar pattern of cleavage products as the 5BSL3.2 wildtype, providing a clear indication that preservation of the 5BSL3.2 structure correlates with viability. It was concluded in [2] that the contrast between C30A-U32G and C30A-U32A suggests that a G in position 32 is capable of altering the upper helix, while an A in position 32 finds no suitable partners within the nucleotides to make up the stem. The functional meaning of the above is that the two-point mutation C30A-U32G alters virus replication (there is a failure of this mutant to replicate) by causing a conformational rearrangement. The G in position 32 is important for this effect to be achieved.

We now experiment with our suggested method for this case. To begin with, the wild-type sequence and its predicted RNAfold optimal structure using a dot-bracket representation is:

AGCGGGGGAGACAUAUAUCACAGCCUGUCUCGUGCCCGACCCCGC

.((((((((((((............))))))........))))))

Next, after running RNAsubopt, it is found that one of the suboptimal solutions, in dot-bracket representation, is:

.((((((..((......))...((......#.*))....))))))

where # is in position 30 in the 5BSL3.2, corresponding to position 84 in the entire 5BSL3, and * is in position 32 in the 5BSL3.2, corresponding to position 86 in the entire 5BSL3. Examining the mutations reported in [2], it is first verified that mutation C30A destabilizes the base pair in the optimal structure and mutation U32G stabilizes the stem in the suboptimal structure, after which we obtain the structure of the two-point mutation C30A-U32G in dot-bracket representation as follows:

.((((((..((......))...((((.....))))....))))))

**Figure 18:**
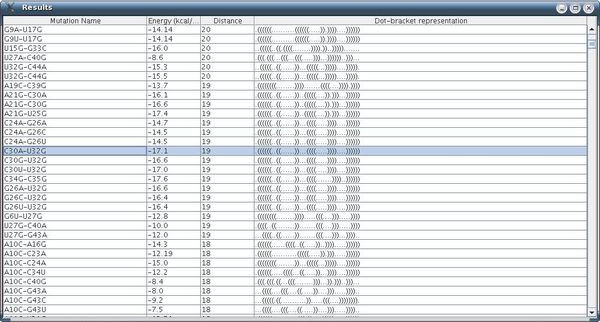
**Mutation Group List Screen for the 5BSL3.2**. Mutation group list screen as a result of our procedure, for the case of 2-point mutations for the 5BSL3.2 wild-type.

**Figure 19:**
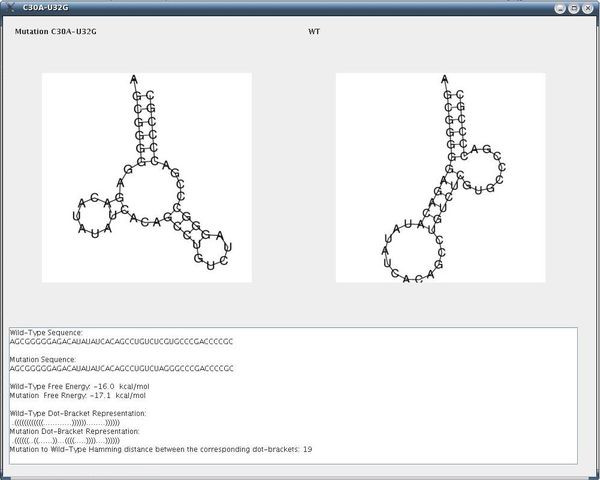
**Output Screen of a Rearranging Mutation in the 5BSL3.2**. Output screen of our procedure for the rearranging mutation C30A-U32G with the secondary structure drawings for the wild-type and the mutant, including additional measures.

In the next step, when running our method with the parameters: dist1 = 12, dist2 = 12, e = 12, numMuts = 2, method = 3 and distance = Hamming we obtain after about 3 minutes on a typical PC the results that appear in Figure 18. By double-clicking on mutation C30A-U32G in the GUI depicted in Figure 18, we obtain the secondary structure drawing that appears in Figure 19, and highlighted is one of the most distant mutations from the wild-type. This mutation corresponds to the mutation from [2], the virus mutant C30A-U32G that fails to replicate, as discussed above. Notably, we find that in full-correspondence with the results and discussion of the experimental results in [2], the two-point mutations in our Figure 18 are from C to A, G, U in position 30 but particularly to G in position 32. This implies that our suggested computational method is in good agreement with the experimental results available in [2].

## Discussion

Considering the possible limitations and the RNA energy minimization prediction methods that we are utilizing and our motivation to use them as accurately as possible, our program is intended to work with sequences no longer than 100–150 nts. Obviously, sequences much longer than 100–150 nts will run into memory problems, but, even more deleterious, in many cases the accuracy of its folding prediction will suffer. For sequences with 200 nts, the value e = 1 or 2 is recommended; for sequences with 150 nts, the value e = 5 is recommended; for sequences with 100 nts, the value e = 10 is recommended, and for shorter sequences of about 70 nts, similar to the examples above, the value e = 15 may be used and a typical PC will return an answer after only a few minutes runtime for sequences with 3-point mutations (RNAsubopt outputs a total number of suboptimal solutions of no more than 6 Mb for e = 15 and a sequence length of 100 nts, and its running time is no more than a few seconds or minutes). In general, a larger e value will provide better results but also a longer running time. For all the cases above, although we obtained a large number of suboptimal secondary structures using RNAsubopt, after running our filters we only remained with about 10–20 different suboptimal solutions. For sequences with 100 – 150 nts or less, RNAsubopt runtime is not a limiting factor if the recommended value of e is used. If the runtime is fast, e can be further increased and another run can be tried. In terms of sequence size and number of point mutations, the actual limitations of our approach are about 200 nts for sequence length and 5-point mutations for the number of point mutations. For longer sequences, prediction reliability starts to decline and the running time will be hours or days instead of minutes, which is also the case for more than 5-point mutations even with a sequence size of about 100–150 nts.

It is possible that some of the mutations found by our approach will not cause much of a conformational rearrangement effect, and their distance from the wild-type solution will be less than dist1. We simply discard such mutations. In addition, some mutations may exhibit not only large distances from their respective wild-type structures, but there may also be large distances between them and the suboptimal solutions from which they were found. Such mutations are printed in the final report, because they are still conformational rearranging mutations.

What is promised by our procedure is that mutations that we find (i.e., all sets of m-point mutations that we find by our procedure) are indeed conformational rearranging mutations that will lead to structures located relatively far from their wild-type secondary structures. Our procedure may miss some mutations that alter the secondary structure of the RNA because we rely strictly on suboptimal solutions as a consequence of energy minimization, and it is possible that there are some mutations that alter the secondary structure but that do not appear in the suboptimal solutions derived by energy minimization. This is possible because the free energy of such a suboptimal solution is very large, and the introduction of a mutation may sufficiently lower the free energy, such that it becomes optimal. Nevertheless, such situations are of the exception, and in practice the method proposed performs accurately and efficiently.

## Conclusion

We present a method that extends RNAmute [18] to treat multiple-point mutations in a tractable manner using suboptimal structures as obtained by Vienna's RNAsubopt [24]. The proposed method is practical, as was demonstrated in two implementation examples. The first with the full P5abc subdomain of the *Tetrahymena thermophila *group I intron ribozyme, showing the success of the efficient approach relative to a 'brute-force' strategy in which all possible multiple-mutations are tried, and explaining the procedure in detail. The second with the 5BSL3.2 sequence of a subgenomic hepatitis C virus (HCV) replicon, showing the success of the suggested computational procedure to predict conformational rearranging mutations that were already found to alter virus replication in a published mutagenesis experiment. The user has the flexibility to choose how much time efficiency is desired vs. how many candidate mutations are to be analyzed. This makes the proposed method particularly suitable for application to specific problems in practice.

## Methods

All calculations performed in this paper were done using the RNAsubopt [24] and RNAfold [11] programs available in the Vienna RNA package version 1.7. The efficient method suggested called MultiRNAmute was compared with a 'brute-force' extension (unpublished) of the original RNAmute [18], which is a straight-forward extension from traversing all single point mutations to traversing all multiple-point mutations without efficiency considerations. For efficiency reasons, the distances between RNA secondary structures are calculated using either a Hamming distance or a base pair distance, depending on the initial choice of the user. Both are implemented with a running time of O(n), where n is the length of the dot-bracket representation of each secondary structure.

## Availability and requirements

**Project name: **MultiRNAmute

**Project home page: **[1]

**Operating system(s): **web access: not applicable, stand-alone: LINUX

**Programming language: **C, Java

**Other requirements: stand alone: **Java 1.4.0 or higher, GNU CC compiler

**License: **None

**Any restrictions to use by non-academics: **None

## Authors' contributions

DB conceived the study, coordinated and participated in software design and drafted the manuscript. AC worked on software design, carried out development and implementation, and participated in drafting the manuscript.

## Supplementary Material

Additional file 1 Supplementary Information on the second filter. Supplementary information on the second filter described in the "Filtering Suboptimal Solutions" subsection of the Results Section. It contains some additional explanations on how it operates, and instructions for the user on how to shut it off. Click here for file
