# Supplementary material for: An efficient method for the prediction of deleterious multiple-point mutations in the secondary structure of RNAs using suboptimal folding solutions
Source: BMC Bioinformatics. 2008 Apr 29;9:222. doi: 10.1186/1471-2105-9-222 (PMC2386494; doi:10.1186/1471-2105-9-222)
Supplement: Additional file 1 — Supplementary Information on the second filter. Supplementary information on the second filter described in the "Filtering Suboptimal Solutions" subsection of the Results Section. It contains some additional explanations on how it operates, and instructions for the user on how to shut it off. [file 1471-2105-9-222-S1.pdf]

Supplementary Information on the Second Filter

The second filter simply adds to the relative stability of each suboptimal secondary structure the number numMuts*3 (3 for base pairs CG or GC, best case for suboptimal) and saves it to a variable called "relSub" that sums up the relative stabilities according to the scores in the weighted Nussinov model described above; the filter also removes the same number numMuts*3 (worst case for the optimal) from the relative stability of the optimal, and saves it to a variable called "relOpt". Finally, the program checks if relSub - relOpt < DIFF, where DIFF is a threshold parameter that is set in the program and controls the desired difference between the relative stabilities of the optimal and suboptimal structures. If relSub - relOpt < DIFF, the filter removes such a suboptimal solution. DIFF is 0 by default because if relSub < relOpt, then the specific suboptimal solution will most likely not become optimal in any case as a consequence of introducing mutations into it. If DIFF is very small, no suboptimal solution will be discarded. Thus, if one sets DIFF = -1000 (found in the src/Multi_Mut.c file in our implementation), the filter is completely shut off. We recommend the value of -1000 in order to shut off the filter since with our simple scoring scheme the highest score for a base pair is 3; given that we are using sequences of length N=200 at most for accurate folding predictions using energy minimization, the lowest value that relSub-relOpt can get to is -300 (3*200 but there are N/2 pairs so divide by 2), and -1000 is significantly lower than that.

To illustrate, using the example provided above the dot bracket representation of the optimal solution of the sequence `UGCCUGCCUCUUGGGAGGGGC` is `.(((..((((....)))))))`, and its relative stability is 20 (there are 6 base pairs CG and 1 AU = 6*3 + 1*2). The relative stability of the suboptimal solution `..((........)).......` is 6. If the number of mutations that we would like to introduce to the sequence is one, the relative stability of this solution after one mutation may increase up to 9 (if the base pair after mutation is CG) and the relative stability of the optimal solution may drop to 17. From this example, we observe that it is impossible for this suboptimal solution to become the optimal solution after a single point mutation because 9 << 17. Therefore, the filter will discard such a suboptimal solution. To summarize, we use this filter only for filtering the suboptimal secondary structures that have no chance of becoming optimal, even after an m-point mutation in the wild-type sequence. The filter can also be shut off by changing the variable DIFF from its default value of 0 to the small negative value of -1000 and re-compiling the code (in our implementation using a makefile).
